# Antiplatelet Therapy for Acute Respiratory Distress Syndrome

**DOI:** 10.3390/biomedicines8070230

**Published:** 2020-07-21

**Authors:** Chuan-Mu Chen, Hsiao-Ching Lu, Yu-Tang Tung, Wei Chen

**Affiliations:** 1 Department of Life Sciences, National Chung Hsing University, 145 Xingda Road, Taichung 402, Taiwan; chchen1@dragon.nchu.edu.tw; 2 The iEGG and Animal Biotechnology Center, and the Rong Hsing Research Center for Translational Medicine, National Chung Hsing University, Taichung 402, Taiwan; 3 Division of Respiratory Therapy, Chia-Yi Christian Hospital, Chiayi 60002, Taiwan; 00614@cych.org.tw; 4 Graduate Institute of Metabolism and Obesity Sciences, Taipei Medical University, Taipei 110, Taiwan; 5 Nutrition Research Center, Taipei Medical University Hospital, Taipei City 110, Taiwan; 6 Cell Physiology and Molecular Image Research Center, Wan Fang Hospital, Taipei Medical University, Taipei 110, Taiwan; 7 Division of Pulmonary and Critical Care Medicine, Chia-Yi Christian Hospital, Chiayi 60002, Taiwan

**Keywords:** acute respiratory distress syndrome, antiplatelet, aspirin, therapy

## Abstract

Acute respiratory distress syndrome (ARDS) is a common and devastating syndrome that contributes to serious morbidities and mortality in critically ill patients. No known pharmacologic therapy is beneficial in the treatment of ARDS, and the only effective management is through a protective lung strategy. Platelets play a crucial role in the pathogenesis of ARDS, and antiplatelet therapy may be a potential medication for ARDS. In this review, we introduce the overall pathogenesis of ARDS, and then focus on platelet-related mechanisms underlying the development of ARDS, including platelet adhesion to the injured vessel wall, platelet-leukocyte-endothelium interactions, platelet-related lipid mediators, and neutrophil extracellular traps. We further summarize antiplatelet therapy, including aspirin, glycoprotein IIb/IIIa receptor antagonists, and P2Y12 inhibitors for ARDS in experimental and clinical studies and a meta-analysis. Novel aspirin-derived agents, aspirin-triggered lipoxin, and aspirin-triggered resolvin D1 are also described here. In this narrative review, we summarize the current knowledge of the role of platelets in the pathogenesis of ARDS, and the potential benefits of antiplatelet therapy for the prevention and treatment of ARDS.

## 1. Introduction

### 1.1. Definition and Epidemiology of ARDS 

Acute respiratory distress syndrome (ARDS), or acute lung injury, is a devastating syndrome that contributes to serious morbidities and mortality in critically ill patients. The definition of this syndrome includes the acute onset of respiratory failure, bilateral infiltrates observed on chest radiographs, hypoxemia with a PaO_2_/FiO_2_ ratio ≤ 300 mmHg with at least positive end-expiratory pressure of 5 cmH_2_O, and no evidence of left atrial hypertension [1,2]. A variety of clinical disorders are associated with the development of ARDS, including direct lung injury, such as bacterial pneumonia, aspiration of gastric contents, or indirect lung injury, such as sepsis, trauma, and transfusion of blood product [3]. The crude incidence of ARDS was around 15 to 80 per 100,000 people per year worldwide [4,5], and the mortality rate is high (approaching 40%) [4]. The only effective management to date to improve the survival rate of this syndrome is a protective lung strategy, with lower tidal volume ventilation [6]. Although numerous promising therapies have been effective in the prevention of ARDS in experimental models, the successful translation to clinical application is still lacking [7,8,9]. In addition, in those who survive the illness, ARDS caused a substantial social burden, such as reduced exercise stamina and health-related quality of life, and increased costs and use of health care services [4,10,11,12,13,14,15]. Therefore, the discovery of medications to prevent the development of ARDS is crucial. 

### 1.2. Pathogenesis of ARDS

**Figure 1:**
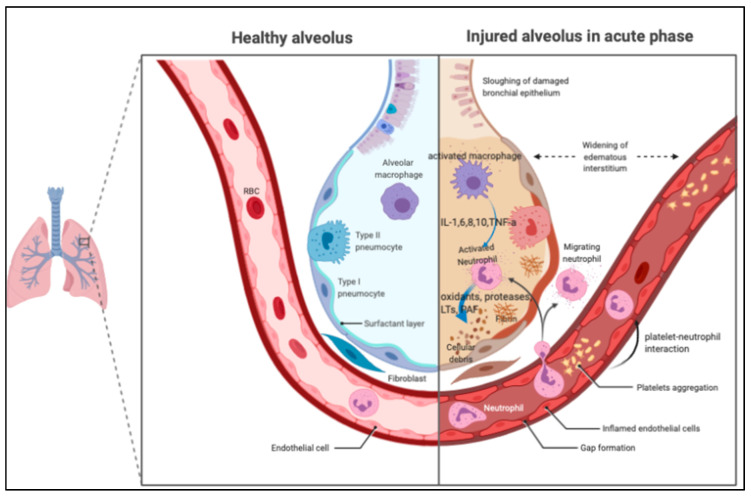
The pathological features of acute respiratory distress syndrome (ARDS) in the acute phase. In the air space, alveolar macrophages secrete cytokines locally to stimulate chemotaxis and activate neutrophils. Neutrophils can release oxidants, proteases, leukotrienes, and other pro-inflammatory molecules, such as platelet-activating factor (PAF). PAF: platelet-activating factor; IL: interleukin; TNF: tumor necrosis factor; LTs: leukotriene.

The pathological features of ARDS are best described at three time points: acute, subacute, and chronic phases [16,17,18]. The acute or exudative phase (the first 1–6 days) is characterized by the influx of protein-rich edema fluid, accompanied by the accumulation of neutrophils, macrophages, exosomes [19], and red blood cells (polymorphonuclear leukocyte [PMN] predominant) into the air spaces as a consequence of the increased permeability of the alveolar–capillary barrier [20,21], as shown in Figure 1. As a result of both endothelial and epithelial injury, denuding of the alveolar epithelium and prominent hyaline membranes can be seen [3,22]. The robust inflammatory response is due to the release of oxidants, proteases, and other potentially toxic agents from activated leukocytes [23,24]. In the air space, alveolar macrophages secrete cytokines, interleukin (IL)-1, -6, -8, and -10, and tumor necrosis factor-α (TNF-α), which act locally to stimulate chemotaxis and activate neutrophils. Macrophages also secrete other cytokines, including IL-1, -6, and -10. IL-1 can also stimulate the production of extracellular matrix by fibroblasts. Neutrophils can release oxidants, proteases, leukotrienes, and other pro-inflammatory molecules, such as platelet-activating factor (PAF). An imbalance between pro-inflammatory and anti-inflammatory mediators can be found in ARDS. A number of anti-inflammatory mediators are also present in the alveolar milieu, including IL-1–receptor antagonist, club cell protein 16 [25], soluble tumor necrosis factor receptor, autoantibodies against IL-8, and cytokines such as IL-10 and -11 [3]. In the subacute or proliferative phase (the next 7–14 days), some edema has usually been reabsorbed, and is often accompanied by interstitial fibrosis, a proliferation of type 2 alveolar cells, and the disruption of capillary function, due to microvascular thrombus formation. Infiltration of fibroblasts and collagen deposition may also be observed. Some experts view ARDS as a vascular microthombotic disease, and its pathogenesis is based on a novel “two-path unifying theory” of hemostasis and “two-activation theory of the endothelium”, promoting molecular pathogenesis [26]. Some patients have a rapid resolution of the disorder [27], but others have progression to fibrotic lung injury, which is called the chronic or fibrotic phase (usually after 14 days). In the chronic phase, there is a resolution of the acute neutrophilic infiltrate with more mononuclear cells and alveolar macrophages in the alveoli, and often more fibrosis, with ongoing evidence of alveolar epithelial repair [17]. 

Overall, dysregulated inflammation [28,29], the inappropriate accumulation and activity of leukocytes and platelets [30], uncontrolled activation of coagulation pathways, and altered permeability of alveolar endothelial and epithelial barriers are pathophysiological hallmarks of ARDS [3,31,32]. Of note, endotheliopathy activates two independent molecular pathways: inflammatory and microthrombotic. The inflammatory pathway initiates inflammation, but the microthrombotic pathway more seriously produces “microthrombi strings” composed of platelet- von Willebrand factor multimer complexes, which become anchored on injured endothelial cells and causes disseminated intravascular microthrombosis [26]. Activated neutrophils affect surrounding lung tissue via several potentially pathogenic cellular mechanisms, including the release of lysosomal proteolytic enzymes [33], the production of prostanoids [34], and the generation of highly reactive oxygen radicals and intermediates [35]. All these mechanisms lead to tissue damage, increased permeability of the alveolar-capillary barrier, and the formation of protein-rich lung edema [36].

## 2. Mechanisms of Platelet in Lung Inflammation 

### 2.1. The Role of Platelet in the ARDS

**Figure 2:**
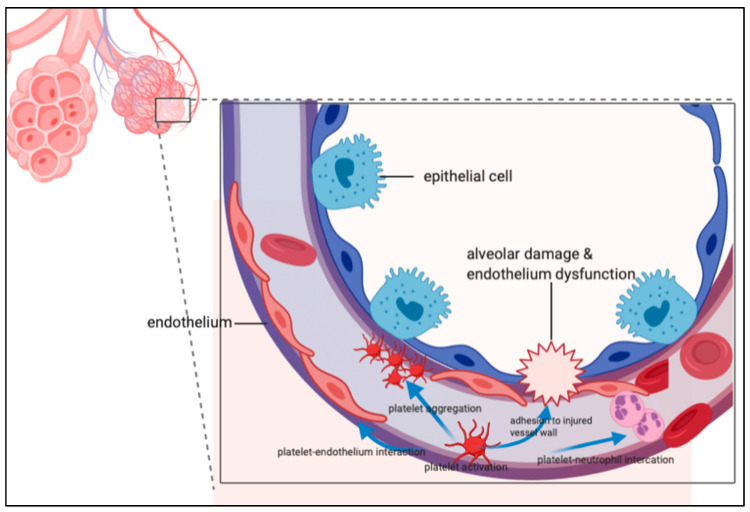
The role of platelets in acute respiratory distress syndrome. Platelets have an increasingly recognized role in the inflammatory response leading to the development of ARDS. The possible mechanisms by which platelets contribute to ARDS include the activation of endothelial cells by the release of pro-inflammatory mediators and adherence of platelets to lung capillary endothelial cells leading to activation of attached leukocytes.

Platelets are anucleated fragments of bone marrow megakaryocytes of approximately 2–4 μm in diameter, and contain glycogen, mitochondria, and at least three types of granules (dense core granules, lysosomes, and α -granules). Following activation, platelets can secrete the content of the granules, change their shape, and upregulate the expression of adhesion molecules including P-selectin, platelet and endothelial cell adhesion molecule 1 (PECAM-1, also known as CD31), glycoprotein (GP) IIb/IIIa (α IIbβ 3) integrin, fibronectin, and thrombospondin [37]. PECAM1 is a member of the immunoglobulin superfamily of adhesion molecules localized at endothelial cell-cell junctions, and contributes to the maintenance of vascular integrity in resting cells, as well as playing a role in the restoration of vascular integrity following barrier disruption [38]. PECAM1 can be cleaved from endothelial cells by a number of mechanisms, including shear stress, resulting in a secreted, shed form of protein (sPECAM1) that is soluble, and can be released into circulation, exerting proinflammatory effects. The upregulation of PECAM1 and/or reducing sPECAM1 through extracorporeal removal or pharmacologic inhibition might be a novel therapeutic strategy in ARDS [39]. Platelets have an increasingly recognized role in the inflammatory response, leading to the development of ARDS. The possible mechanisms by which platelets contribute to ARDS include the activation of endothelial cells by the release of pro-inflammatory mediators [40,41,42,43,44,45,46] and adherence of platelets to lung capillary endothelial cells leading to the activation of attached leukocytes [47], as shown in Figure 2. 

### 2.2. Platelet Adhesion to the Injured Vessel Wall

Not only neutrophils, but also platelets, are shown to adhere to injured capillary endothelium in the acute phase of ARDS. At sites of vascular injury and endothelial denudation, platelets adhere to activated endothelial cells or in the subendothelial matrix [48] directly or indirectly. The direct binding of platelets to extracellular collagen at sites of vascular injury is mediated via several GPs. Platelet surface receptors GP Ia/IIa, GPIV, and GPVI interact directly with collagen, and platelet surface receptors GP Ib/V/IX interact with von Willebrand factor [49]. Binding the GP Ib/IX/V complex to von Willebrand factor initiates the activation of the integrin αIIbβ3 on platelets, resulting in platelet aggregates via RGD-containing bridging molecules, such as fibrinogen, fibronectin, and thrombospondin [50,51,52]. In addition, the platelet receptor C-type lectin-like 2 (CLEC-2) has been shown to regulate vascular integrity at sites of acute inflammation [53]. CLEC-2 expressed in platelets is required to limit neutrophil recruitment, which, in turn, limits lung function decline in a mouse model of ARDS. In addition, the expression of the CLEC-2 ligand podoplanin is required in hematopoietic cells to limit neutrophil chemokine expression and, consequently, arterial oxygen saturation decline [54]. 

### 2.3. Platelet-Leukocyte-Endothelium Interactions

**Figure 3:**
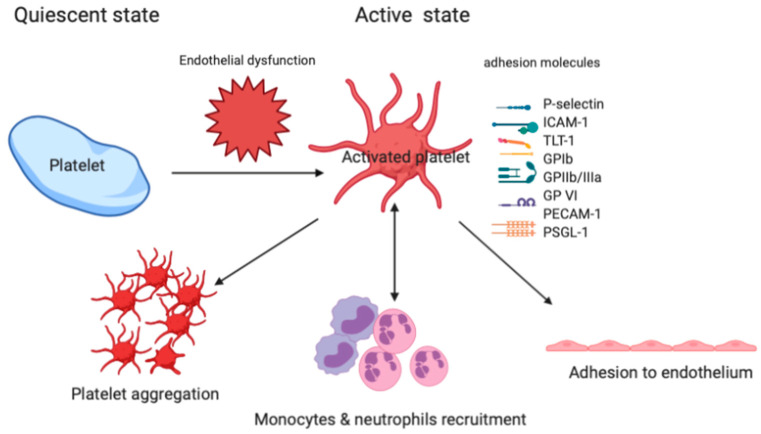
The role of specific adhesion molecules in the platelet-neutrophil interaction, adhesion to endothelium, and platelet aggregation. Triggering receptor expressed on myeloid cells (TREM)-like transcript-1: TLT-1; intercellular adhesion molecule-1: ICAM-1; P-selectin glycoprotein ligand 1: PSGL-1; glycoprotein: GP; Platelet and endothelial cell adhesion molecule-1: PECAM-1.

Platelet-neutrophil-endothelium interactions are involved in the lung inflammation of ARDS. As shown in Figure 3, platelet adhesion to the intact endothelium is mediated by at least two different types of adhesion molecules, selectins and integrins. GP IIb/IIIa (αIIbβ3 integrin) is an important adhesion molecule in platelets that is responsible for mediating platelet aggregation and some platelet-neutrophil-interactions. Injection of platelet-specific monoclonal antibodies against the GP IIb/IIIa receptor in mice causes early signs of acute lung injury with increased cellularity in the lung interstitium and rapid engorgement of alveolar septal vessels [55]. P-selectin, a type I membrane protein, is stored in the α-granules of platelets and Weibel-Palade bodies of endothelial cells, from where it is rapidly expressed on the cell surface by a Ca^2+^-dependent translocation to the plasma membrane [56]. Upon activation, platelets bind to endothelial cells by expressing P-selectin via GP Ibα and P-selectin glycoprotein ligand-1 (PSGL-1). Following a conformational change, αIIbβ3 integrins establish strong adhesion of platelets by binding to small bridging ligands, such as fibrinogen or vitronectin, which interact with the endothelial adhesion molecules ανβ3 or intercellular adhesion molecule 1 (ICAM-1) [57]. To attract the neutrophils, P-selectin mediates the initial binding (‘capturing”) of platelets to leukocytes and leukocytes to endothelial cells. Following the adhesion of neutrophils to platelets, neutrophils are activated through PSGL-1 [58], the triggering receptor expressed on myeloid cells (TREM)-1 [59], lipid mediators, and chemokines presented by platelets. In an experimental study, Zhao et al. showed that the blockade of PSGL-1 results in diminished alveolar neutrophil transmigration in lipopolysaccharide (LPS)-induced acute lung injury in mice, indicating that platelets and their interaction with neutrophils are requisite for the development of LPS-induced lung inflammation and injury [60]. TREM-like transcript-1 (TLT-1) is a type-1 immunoglobulin domain receptor that is stored in platelet α-granules and, upon platelet activation, translocate to the surface. TLT-1 uses fibrinogen to govern the transition between inflammation and hemostasis, and facilitates controlled leukocyte transmigration during the progression of ARDS [61]. Collectively, activated platelets play a crucial role in host defense, influencing pulmonary neutrophil recruitment, and contribute to the development of ARDS [40,62,63]. Upon activation, platelets can adhere to other platelets or leukocytes, forming neutrophil platelet aggregates (NPAs) or monocyte-platelet aggregates (MPAs), and to exposed endothelium at sites of inflammation [64]. The presence of circulating leukocyte platelet aggregates (LPAs) is a sensitive indicator of platelet activation, and thrombus formation has been associated with the severity of acute lung injury [65]. Furthermore, Ortiz-Muñoz et al. reported that the dynamic formation of LPAs observed by using lung intravital microscopy sharply increased with acute lung injury [66]. The measurement of LPA in patients with ARDS and other critical illnesses could be a useful biomarker of inflammation, and could be measured serially, to assess therapeutic responses to treatment with pro-resolving lipid mediators.

### 2.4. Lipid Mediators and Platelet Aggregation

The aggregation of platelets at sites of lung injury to facilitate the recruitment of neutrophils to the injured alveolus is an important mechanism in the development of ARDS [40]. Lipid mediators play a pivotal role in the aggregation of platelets and regulation of inflammation, and arachidonic acid-derived products, such as thromboxane (TX) and leukotrienes (LTs), have been implicated as pro-inflammatory mediators of ARDS [67]. For aggregation, platelets release arachidonic acid through a membrane-bound lipase. Arachidonic acid is converted to TXA2 via a multistep process, which is a powerful mediator of platelet aggregating response. Cyclooxygenase is the key enzyme responsible for the synthesis of prostaglandins (PGs) and TXA2 in platelets. In addition, cyclooxygenase is involved in oxidative stress-induced acute lung injury, suggesting a link between neutrophil-derived oxidative stress and endothelial eicosanoid metabolism [68]. Aspirin has significant antiplatelet properties through the inhibition of cyclooxygenase enzymes that prevent TXA2 production, therefore suppressing platelet aggregation in animal models of acute lung injury [69]. 

*Cytosolic phospholipase A2 (cPLA2)* is a key enzyme for the production of inflammatory mediators, such as TXs and LTs, which are generated from arachidonic acid by cyclooxygenase and 5-lipoxygenase, respectively. Nagase et al. reported that the disruption of the gene encoding cPLA2 significantly reduced pulmonary edema, PMN sequestration, and deterioration of the gas exchange in a murine model of LPS-induced acute lung injury [70], indicating that the inhibition of cPLA2-initiated pathways may provide a therapeutic approach to acute lung injury. On the contrary, cPLA2 could act with the reactive oxygen species produced during intestinal ischemia-reperfusion, resulting in the exacerbation of the inflammatory reaction in ARDS [71]. Platelet-activating factor (PAF), a potent phospholipid activator and one of the lipid mediators of platelet aggregation, is also associated with the development of ARDS [72]. The presence of G994T polymorphism in exon 9 of the plasma PAF acetylhydrolase gene has a better survival rate in ARDS [73]. 

### 2.5. Neutrophil Extracellular Traps (NETs)

Sepsis syndrome is the primary etiology of ARDS and is associated with a 35–45% incidence of ARDS development [74]. It has been hypothesized that endotoxemia and phagocytosis of bacteria are involved in the pathogenesis of septic syndrome-associated ARDS [75]. Platelets express toll-like receptors (TLRs), including TLR2 and TLR4, that recognize the common bacterial molecules peptidoglycan and LPS, respectively [76]. Activated platelets, particularly in the context of LPS stimulation, trigger the release of extracellular DNA traps (NETs), with proteolytic activity from neutrophils, serving to capture and degrade microbes [76]. These NETs are capable of trapping and killing extracellular pathogens in blood and tissues during infection [77]. However, NETs are not only produced during severe infections, but have also been observed in various inflammatory diseases [78,79,80]. Caudrillier et al. showed that platelet-induced NETs contribute to lung endothelial injury, and that targeting NET formation with either aspirin or a GP IIb/IIIa inhibitor decreased NET formation and lung injury in the experimental model of transfusion-related acute lung injury (TRALI) [62]. Nitrostyrene derivatives (BNSDs) have been identified as inhibitors of phospholipase and tyrosine kinase, antibacterial agents, and macrophage immune response regulators, and attenuate LPS-mediated acute lung injury via the inhibition of neutrophil-platelet interactions and NET release [81]. 

## 3. Antiplatelet Agents in Experimental Studies

### 3.1. Aspirin

Aspirin is a well-known, irreversible, noncompetitive inhibitor of arachidonic acid cyclooxygenase metabolism and is commonly used in clinical practice. Preclinical studies have shown that aspirin can prevent or treat ARDS by decreasing neutrophil activation and recruitment to the lung, TNF-α expression in pulmonary intravascular macrophages, plasma TX B2 levels, and platelet sequestration in the lungs [62,69,82,83,84,85]. Aspirin also reduces the severity of edema and vascular permeability in oxidative stress-induced acute lung injury [68]. Looney et al. showed that treatment with aspirin prevented lung injury and mortality, but blocking P-selectin or CD11b/CD18 pathways did not. These data suggest a 2-step mechanism of TRALI: priming hematopoietic cells, followed by vascular deposition of activated neutrophils and platelets that then mediate severe lung injury [69]. In addition, Bates et al. showed that delayed postoperative neutrophil apoptosis is significantly preserved in patients taking 300 mg of aspirin on the day before surgery, indicating that aspirin may be able to ameliorate to promote a resolution for persistent inflammation [86].

Another function of aspirin in treating acute lung injury is the acetylation of cyclooxygenase-2 (COX-2) that causes a conformational change, leading to the inhibition of prostanoid synthesis [87]. The acetylation of COX-2 switches catalytic activity to convert arachidonic acid to 15R-hydroxyeicosatetraenoic acid, which can be subsequently converted to 15(R)-epi-lipoxin A4 (15[R]-epi-LXA4), also known as aspirin-triggered lipoxin (ATL) [88]. Lipoxins are endogenous lipid mediators generated during inflammation that can block inflammatory cell recruitment, inhibit cytokine release, and decrease vascular permeability, which collectively are anti-inflammatory properties [89,90]. Ortiz-Muñoz et al. showed that aspirin treatment increased levels of ATL, and treatment with ATL in both lipopolysaccharide and TRALI models protected the lung from acute lung injury [66]. In addition, delayed neutrophil apoptosis is a prominent feature of ARDS [91], which results in prolonging the period of lung injury and hypoxia. Aspirin has previously been shown to preserve neutrophil apoptosis [86], and experimental evidence suggests that ATL restores neutrophil apoptosis and enhances the resolution of alveolar inflammation [92].

**Figure 4:**
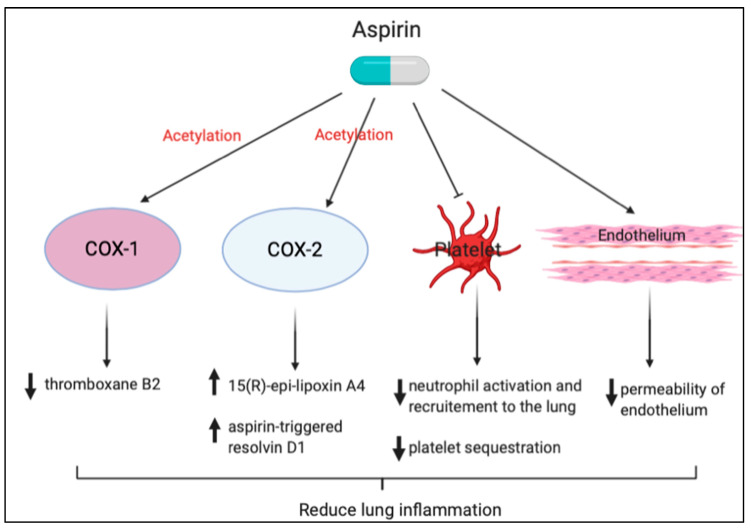
Protective effects of aspirin against lung inflammation. Neutrophil recruitment to the sites of lung injury may also be modulated through aspirin-triggered anti-inflammatory mediators.

Neutrophil recruitment to sites of lung injury may also be modulated through aspirin-triggered anti-inflammatory mediators. In the case of aspirin treatment, aspirin-acetylated COX-2 generates 17R-HDHA, which, following sequential oxygenation by 5-lipoxygenase, results in the production of 17-epi-RvD1, also known as aspirin-triggered RvD1 (AT-RvD1). AT-RvD1 is the 17R epimer of RvD1 (7S, 8R, 17R-trihydroxy-4Z, 9E, 11E, 13Z,15E, 19Z-docosahexaenoic acid), which is more resistant to catalysis than RvD1 [93]. Eickmeier et al. showed that AT-RvD1 inhibited neutrophil-platelet heterotypic interactions by downregulating both P-selectin and its ligand CD24. AT-RvD1 also significantly decreased levels of bronchoalveolar lavage fluid pro-inflammatory cytokines, including IL-1β, IL-6, and TNF-α, and decreased nuclear factor-κB (NF-κB)-phosphorylated p65 nuclear translocation in an acid-initiated lung injury model. This suggests that aspirin therapy might decrease the severity and augment the resolution of ARDS [85]. Tang et al. demonstrated, for the first time, that AT-RvD1– and p-RvD1-treated mice have significantly reduced lung inflammatory responses, including TNF-α, IL-6, keratinocyte cell-derived chemokine, and macrophage inflammatory protein (MIP)-1α and reduced lung injury after immunoglobulin G immune complex deposition, suggesting a new approach to blocking immune complex-induced inflammation [94]. Our study also showed that pretreatment with aspirin reduced NF-κB activation, active oxygen species expression, the number of macrophages, neutrophil infiltration, and lung edema compared with hyperoxia-only treatment in NF-κB-luciferase transgenic mice [95]. Cox et al. showed that AT-RvD1 treatment resulted in reduced oxidative stress, increased glutathione production, and significantly decreased tissue inflammation, indicating that AT-RvD1 is an effective therapy for prolonged hyperoxic exposure in this murine model [96]. A brief summary of the aspirin effect is shown in Figure 4.

### 3.2. GP IIb/IIIa Antagonists

GP IIb/IIIa receptor inhibitors that mediate platelet-platelet binding through fibrinogen [97] are currently used as a preventive medication for coronary artery disease after percutaneous coronary intervention. Commercially available GP IIb/IIIa receptor inhibitors include abciximab, eptifibatide, and tirofiban.

In a murine model of influenza A virus infections, GP IIb/IIIa antagonist, eptifibatide is shown to protect mice from death caused by influenza viruses, by reducing aggregates of activated platelets [98]. Sharron showed that eptifibatide attenuates platelet granzyme B-mediated apoptosis and results in less severe sepsis and extended survival in a murine model of abdominal sepsis [99], indicating that the GP IIb/IIIa antagonist may be a target for the treatment of sepsis-related ARDS. Caudrillier et al. also showed that another GP IIb/IIIa antagonist, tirofiban, was shown to be effective in the treatment of TRALI by decreasing soluble NET components [62].

### 3.3. P2Y12 Inhibitors

The P2Y12 protein, a chemoreceptor for adenosine diphosphate, is found mainly but not exclusively on the surface of platelets and belongs to the Gi class of a group of G protein-coupled purinergic receptors [100]. Commercially available P2Y12 inhibitors include clopidogrel, prasugrel, and ticagrelor, mainly indicated for cardiovascular diseases. Several studies have shown that clopidogrel not only diminishes the risk of atherothrombotic events, but also reduces the markers of systemic inflammation, including C-reactive protein, soluble CD62P (P-selectin) and CD54, pro-inflammatory cytokines, and platelet-leukocyte conjugates [101]. Harr et al. showed that rats pretreated with clopidogrel were protected from trauma/hemorrhagic shock-related acute lung injury by reducing platelet activation and aggregation, microthrombi formation, and leukocyte recruitment [102]. Le et al. also showed that clopidogrel had a similar effect as the GP IIb/IIIa antagonist, to protect mice from death caused in an experimental model of influenza virus A infection [98]. Tuinman et al. reported that clopidogrel attenuates LPS-induced lung injury in mice, but its effect is inferior to high-dose aspirin [83].

For antiplatelet therapy to treat or prevent ARDS, there are several major differences between animal experiments and human studies. First, the lung injury model of animal experiments cannot fully comply with the process of human acute lung injury. Second, people who develop lung injury usually have comorbidities, which can lead to different outcomes even with the same treatment. Third, the therapeutic dose for animals, once used in humans, may be too high and cause side effects. Fourth, there are too many causes for ARDS in the human body, which results in so-called heterogeneity. For these reasons, it is quite difficult for a single drug to be successful in people with ARDS.

## 4. Antiplatelet Agents in Clinical Studies

The human study regarding aspirin for treating or preventing ARDS was conducted around 20 years ago. As shown in Table 1, Erlich and colleagues performed a retrospective analysis of 161 patients from Olmsted County, Minnesota, to assess a potential association between prehospital use of antiplatelet agents and the development of ARDS in at-risk patients. Antiplatelet agents included aspirin, clopidogrel, and ticlopidine in their study. They showed that antiplatelet therapy was associated with a reduced incidence of ARDS (12.7% vs 28.0%; OR, 0.37; 95% CI, 0.16–0.84; *p* = 0.02), even after adjusting for confounding variables [103]. Meanwhile, O’Neal et al., from our laboratory, conducted a secondary analysis of a prospective study with 575 patients in the validating acute lung injury markers for diagnosis (VALID) cohort, and showed that concurrent statin and aspirin use, but not aspirin alone, was associated with the reduced risk of ARDS [104]. However, this study was likely underpowered to show an independent association between prehospital aspirin use and the reduced risk of ARDS, given the large proportion of patients who were receiving both prehospital statin and prehospital aspirin therapy. Later, Kor et al., from a group of lung injury prevention study investigators (USCIITG–LIPS), performed a multicenter prospective observational study to evaluate the association between prehospitalization aspirin therapy and incident acute lung injury in a heterogeneous cohort of at-risk medical patients. In total, 3855 at-risk patients were enrolled from 22 hospitals over a 6-month period in the United States and Turkey. Nine hundred seventy-six (25.3%) were receiving aspirin at the time of hospitalization. Two hundred forty (6.2%) patients developed acute lung injury. After adjusting for the propensity to receive aspirin therapy, they found that no statistically significant associations between prehospitalization aspirin therapy and ARDS [105]. However, the overall incidence of ARDS in their study was low (6.2%). To further characterize the possible benefit of prehospital aspirin use in ARDS, we performed a new cross-sectional analysis of the entire prospectively collected VALID cohort, with approximately 1149 critically ill patients (age ≥ 40) enrolled during a 6-year interval. Our data showed that patients with prehospital aspirin had a significantly lower incidence of ARDS (27% vs. 34%, p = 0.034). In a multivariable, propensity-adjusted analysis, including age, gender, race, sepsis, and APACHE II, prehospital aspirin use was associated with a decreased risk of ARDS (OR 0.66, 95% CI 0.46–0.94) in the entire cohort, and a subgroup of 725 patients with sepsis (OR 0.60, 95% CI 0.41–0.90) [106]. In a prospective study of 202 patients with ARDS, aspirin therapy, given either before or during the hospital stay, was associated with a reduction in ICU mortality (OR: 0.38, CI: 0.15–0.96, p = 0.04) by using multivariate logistic regression analysis [107].

A group of the lung injury prevention study with aspirin (LIPS-A) conducted a multicenter, double-blind, randomized clinical trial, testing whether early administration of aspirin would result in a reduced incidence of ARDS in adult patients at high risk. Approximately 400 participants from 14 hospitals across the United States were enrolled [108]. The results showed that, among at-risk patients presenting to the emergency department, the use of aspirin compared with placebo did not reduce the risk of ARDS at 7 days [109]. However, there were several limitations in this study, including an unexpectedly lower rate of ARDS, a large number of patients who used antiplatelets had been excluded, the time from randomization to first drug administration was longer than anticipated at study onset, and the aspirin dose chosen for this study was too low [109]. Furthermore, most studies did not have a subgroup analysis. ARDS is caused by a variety of etiologies, and we do not know whether aspirin is only beneficial for certain causes. Toner et al. suggested that aspirin can modulate multiple pathogenic mechanisms implicated in the development of sepsis-related ARDS [110]. A bold study that recruited healthy volunteers to receive either 75 or 1200 mg aspirin for 7 days prior to LPS inhalation showed that aspirin reduced pulmonary neutrophilia and tissue-damaging neutrophil proteases (matrix metalloproteinase (MMP)-8/-9), reduced bronchoalveolar lavage concentrations of TNF-α, and reduced systemic and pulmonary TXB2 [45]. 

## 5. Meta-Analysis of Clinical Studies 

As shown in Table 2, Panka et al. selected 15 pre-clinical studies and eight clinical studies, and showed that aspirin plays a beneficial role in ARDS prevention and treatment [44]. Yu et al. reviewed six studies and showed aspirin could reduce the rate of ARDS/ALI (OR:0.71) but not the mortality [111]. Jin et al. reviewed seven studies and showed significantly lower odds of ARDS in the prehospital antiplatelet therapy group, compared with subjects with no prehospital antiplatelet therapy (odds ratio: 0.68) [112]. Mohananey et al. included 17 studies, and found that there was a significant reduction in all-cause mortality in patients on antiplatelet therapy, compared to the control (OR: 0.83). Both the incidence of ARDS (OR: 0.67) and the need for mechanical ventilation (OR: 0.74) were lower in the antiplatelet group [113]. On the contrary, Wang et al. analyzed nine eligible studies, and found that antiplatelet therapy did not significantly decrease hospital mortality in high-risk patients, and an association with the incidence of ARDS remains unclear [43].

In summary, it is still unknown whether aspirin can be used to effectively prevent or treat ARDS. This can be attributed to several factors. First, there is considerable heterogeneity in ARDS itself. Several recent research directions are exploring whether different treatments can be given according to different phenotypes of ARDS [114,115]. Therefore, future research should be based on ARDS patients whose phenotype pathology is more aligned with the mechanisms of aspirin. Second, for previous studies, the starting point of aspirin administration, the dose administered, and the duration of administration are all very different. This is also one of the reasons for the inconsistency of the research results. Therefore, whether future research can detect some biomarkers to track their drug response is quite important. Third, even if the above two reasons are resolved, the mechanism of aspirin’s action is only a part of the treatment or prevention of ARDS. Perhaps a cocktail therapy, which can combine aspirin with certain medications, would be beneficial for the prevention or treatment of ARDS.

## 6. Conclusions

In summary, platelets play a crucial role in the pathogenesis of ARDS in a number of experimental studies, and antiplatelet therapy exerts a potential therapeutic benefit for ARDS in clinical studies. The main effects of antiplatelet therapy to reduce the severity of ARDS are possibly based on four directions: (1) reducing platelet adhesion to the injured vessel wall; (2) inhibiting platelet-leukocyte-endothelium interactions; (3) modulating lipid mediator related platelet aggregation, and (4) inhibiting NETs formation. Several observational studies have shown that aspirin is protective against the development of ARDS, and a large multicenter, double-blinded, randomized study showed no beneficial effect of aspirin on the development of ARDS. Future research should be based on ARDS patients whose phenotype pathology is more aligned with the mechanisms of antiplatelet therapy, and specific biomarkers should be developed to track their drug response. 

## Figures and Tables

**Table 1:** A summary of recent cohort studies into antiplatelet therapy for ARDS.

Authors	Study Country	Study Design	Patient’s Inclusion Criteria	Patient No.	Medications	Dosage (mg)	Medication Given Time Point	Outcome Variables	Results
Erlich el al. [103]	Minnesota, US	Retrospective cohort	at least one major risk factor for ALI & age > 18 years	161	Any kind of antiplatelet medications		at the time of hospital admission	development of ALI or ARDS ICU and hospital mortality	Reduced incidence of ALI/ARDS (OR:0.37)
O’Neal Jr et al. [104]	Tennessee, US	Prospective cohort	critically ill patients admitted to the medical or surgical ICU and age >18 years	575	Aspirin combined with statin	81or 365 daily	Prehospital use	Development of severe sepsis, ARDS Hospital mortality	Two combined had the lowest rates of severe sepsis, ALI/ARDS and mortality.
Kor et al. [105]	20 hospitals in US & 2 in Turkey	Prospective cohort	non-surgical patients admitted to the hospital with at least one major risk factor for ALI and age >18 years	3855	Aspirin	No mention	at the time of hospital admission	development of ALI or ARDS ICU and hospital mortality	No significant effect
Chen et al. [106]	Tennessee, US	Prospective cohort	critically ill patients (age ≥ 40) admitted to the medical or surgical ICU	1149	Aspirin	81 or 365 daily	Prehospital use	Development of ARDS Development of sepsis related ARDS	Decreased risk of ARDS (OR: 0.66) in the entire cohort also sepsis (OR: 0.60)
Boyle et al. [107]	United Kingdom	Prospective cohort	patients (>16 years-old) requiring invasive mechanical ventilation admitted to the medical or surgical ICU	202	Aspirin	No mention	both pre-admission and during ICU stay	ICU mortality	Reduction in ICU mortality (OR: 0.38)
Kor et al. [109]	16 US academic hospitals	multicenter, double-blind, RCT	patients at risk for ARDS (Lung Injury Prediction Score ≥4)	7673	Aspirin	325 loading dose followed by 81	within 24 hours of emergency department presentation	Development of ARDS by study day 7 Hospital length of stay Biomarkers	No significant effect
Hamid et al. [45]	United Kingdom	double-blind, RCT	Healthy volunteers	33	Aspirin	75 or 1200 for 7 days	seven days prior to LPS inhalation	Inflammatory biomarkers	Reduced pulmonary neutrophilia and tissue damaging, reduced BAL concentrations of TNF-α and pulmonary TXB2.

US: United States; RCT: randomized controlled trial; ALI: acute lung injury; ARDS: acute respiratory distress syndrome; BAL: bronchoalveolar lavage; TNF: tumor necrosis factor; TX: thromboxane; OR: odds ratio.

**Table 2:** Summary of meta-analysis of the effect of aspirin on ARDS.

Authors	Enrolled Types of Studies	Enrolled Study Numbers	Medications	Outcome Variables	Results	Between-Study Heterogeneity	Conclusions
Panka et al. [44]	1 RCT, 7 OS	8	Aspirin	the risk of ARDS	pooled OR was 0.59	Q = 2.44, I^2^ = 68%	A beneficial role for Aspirin in ARDS prevention and treatment.
Yu et al. [111]	1 RCT, 5 OS	6	Aspirin	the risk of ARDS/ALI mortality.	pooled OR was 0.71	I^2^ = 0%, P = 0.419	Aspirin could provide protective effect on the rate of ARDS/ALI, but it could not reduce the mortality.
Jin et al. [112]	7 OS	7	Antiplatelet agents (aspirin 75 to 300 mg daily), (clopidogrel, 75 mg daily), and ticlo- pidine.	the risk of ARDS/ALI mortality.	pooled OR was 0.68	I^2^ = 34%	Pre-hospital antiplatelet therapy was associated with a reduced rate of ARDS but had no effect on the mortality in the subjects at high risk
Mohanney et al. [113]	17 OS	17	Aspirin and other antiplatelet agents	the risk of ARDS/ALI mortality.	pooled OR was 0.67	I^2^ = 25%	Antiplatelet therapy had an improved survival, decreased incidence of ARDS
Wang et al. [43]	2 RCT, 7 OS	9	Aspirin and other antiplatelet agents	the risk of ARDS/ALI mortality.	pool OR was 0.68 from OS; but no significant difference in RCT	*I^2^* = 0.0%, *p* = 0.329 for RCT *I*^2^ = 68.4%, *p* = 0.004 for OS	Whether antiplatelet therapy is associated with a decreased incidence of ARDS in patients at a high risk of developing the condition remains unclear.
